# Nuclear expansion and pore opening are instant signs of neuronal hypoxia and can identify poorly fixed brains

**DOI:** 10.1038/s41598-018-32878-1

**Published:** 2018-10-03

**Authors:** Anisa Dehghani, Hulya Karatas, Alp Can, Esra Erdemli, Muge Yemisci, Emine Eren-Kocak, Turgay Dalkara

**Affiliations:** 1 Hacettepe University, Institute of Neurological Sciences and Psychiatry, Ankara, 06100 Turkey; 2 Ankara University, School of Medicine, Department of Histology and Embryology, Ankara, 06100 Turkey; 3 Hacettepe University, Faculty of Medicine, Department of Neurology, Ankara, 06100 Turkey; 4 Hacettepe University, Faculty of Medicine, Department of Psychiatry, Ankara, 06100 Turkey

## Abstract

The initial phase of neuronal death is not well characterized. Here, we show that expansion of the nuclear membrane without losing its integrity along with peripheralization of chromatin are immediate signs of neuronal injury. Importantly, these changes can be identified with commonly used nuclear stains and used as markers of poor perfusion-fixation. Although frozen sections are widely used, no markers are available to ensure that the observed changes were not confounded by perfusion-induced hypoxia/ischemia. Moreover, HMGB1 was immediately released and p53 translocated to mitochondria in hypoxic/ischemic neurons, whereas nuclear pore complex inhibitors prevented the nuclear changes, identifying novel neuroprotection targets.

## Introduction

Cell death after ischemia, hypoxia or cardiac arrest is an extensively investigated subject^1–5^. To our knowledge, the earliest time point examined in *in vivo* studies is usually 15 minutes after the ictus^1,6^. According to these studies, the first histological changes emerging are astrocyte endfeet swelling as well as neuronal shrinkage or swelling^1,2,7–9^. These well-established features are used to identify and eliminate poorly perfused-fixed brains when examining paraffin-embedded tissue sections. Unfortunately, there are no such standards for recognizing inadequately perfused-fixed frozen sections, in which fine cellular structural details cannot be unambiguously detected. Given that frozen sections are increasingly preferred in experimental neuroscience to be able to utilize florescent-tagged antibodies and probes or transgenic animals expressing florescent proteins^10–12^, there is a pressing need for markers of delayed fixation in frozen sections to ensure that the observed changes were not due to non-optimal tissue perfusion, causing brief hypoxia/ischemia before complete tissue fixation. Therefore, identification of the immediate histological changes induced by hypoperfusion/hypoxia may not only disclose the events taking place within the first few minutes of ischemic/hypoxic neuronal injury and identify novel neuroprotection targets but may also help recognizing poorly perfused-fixed brain sections. While identification of the early targets is critical for preventive neuroprotection, detecting the markers of poor perfusion on frozen section is also of wide interest to neuroscientists.

The goal of fixation via transcardial perfusion is to preserve the tissue uniformly in a life-like state without allowing hypoxia/hypoperfusion-induced changes while sacrificing the animal. Perfusing the fixative directly by way of circulatory system delivers the fixative rapidly and efficiently throughout the body unlike immersing the extracted tissue in a fixative solution. Flushing out the blood is another advantage of this method, decreasing non-specific immunostaining of serum immunoglobulins. However, due to technical difficulties (e.g. improper localization of intraventricular needle or ineffective heart contractions), it is not uncommon that perfusion pressure may not be optimal and the brain tissue cannot be rapidly fixed although the peripheral signs of good perfusion such as whitening of the cornea, twitching and stiffening of the muscles and tail are observed with aldehydes.

Here, we investigated the cellular changes emerging within a few minutes during hypoperfusion/hypoxia due to inadequate intravascular perfusion pressure and hypoxia. We found that swelling of the neuronal nuclei without losing the integrity of nuclear envelope along with chromatin margination are the earliest histological changes that can identify injured neurons. We show that peripheral staining of swollen nuclei creating a donut-like image with commonly used nuclear stains such as Hoechst on frozen sections can easily identify poorly-perfused brains. Moreover, expansion of nuclear membrane with chromatin margination appears to be a very early sign of neuronal compromise that has not been widely recognized^13^ and, intriguingly, can be prevented by inhibitors of nuclear pore complex, disclosing a new drug target for neuroprotection.

## Results

### Markers of instantaneous neuronal injury on poorly perfused-fixed frozen sections

**Figure 1:**
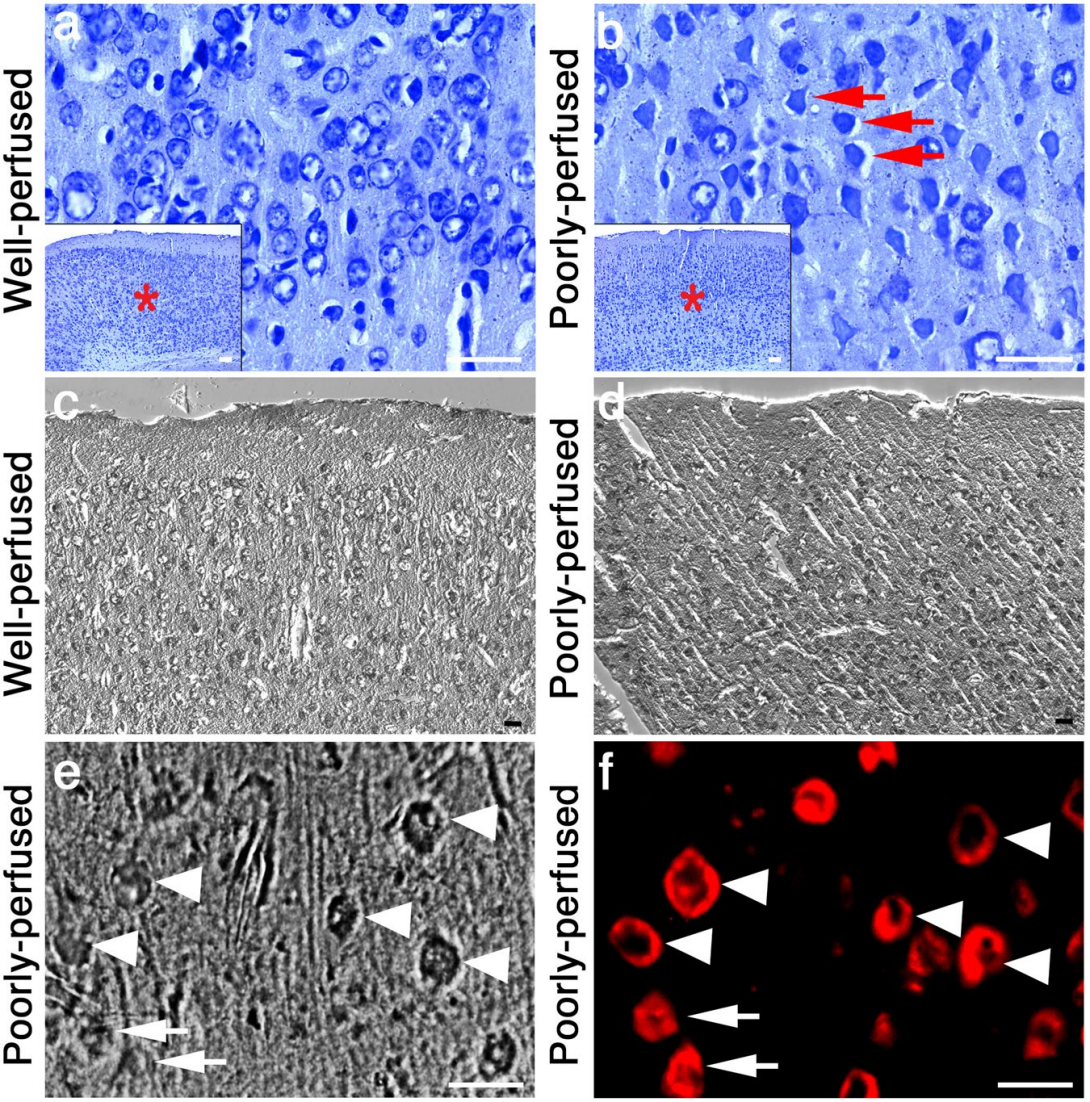
Dysmorphic neurons surrounded by swollen astrocyte end-feet displayed a donut-like staining with NeuN. Unlike well-perfused Nissl-stained paraffin-embedded brain sections (**a**), swollen astrocyte end-feet (AEF) surrounding the soma of dysmorphic neurons were observed in poorly-perfused sections (arrows in **b**) as typically seen after hypoxia/ischemia. DIC images (**c** and **d**) showed that, in contrast to granular surface corresponding to cell bodies and capillaries in well-perfused sections (**c**), a striated tissue surface as a common type of artifact was noted in poorly-perfused cortical sections consistent with cellular edema (**d**). NeuN immunostaining of the frozen brain sections confirmed that the dysmorphic cells were neurons (arrowheads in **f**) surrounded by swollen cellular processes (AEF) as illustrated by phase contrast imaging of the same section (low contrast rim - arrowheads in **e**). Intriguingly, these dysmorphic neurons displayed a circular NeuN staining pattern (donut-like stainings - arrowhaeds in **f**), leaving the center part of the nucleus unstained. Intact neurons in poorly perfused sections exhibited a homogenous NeuN labeling and did not show the pericellular low contrast rim in phase contrast microscopy (arrow in **e** and **f**). Nissl-stained and DIC images were taken from cortical areas 4 (*). Scale bars: 25 µm.

During poor perfusion, neurons are subjected to hypoxic/ischemic injury until they are completely fixed. To search for very early indicators of this neuronal injury as potential fluorescent markers of the poor perfusion in frozen sections, we divided the perfused brain into two and processed one half for frozen sections and the other half for paraffin-embedded sections. The well-characterized histopathological changes induced by hypoperfusion/hypoxia for paraffin sections, verified that the brain examined was indeed poorly perfused. In paraffin sections, degenerating neurons displaying early ischemic/hypoxic changes were visible with Nissl staining unlike the well-fixed ones. These sections also exhibited the typical astrocyte end-feet swelling around dysmorphic neurons^1,6,8,9^ (Fig. 1a,b). When the same Nissl-stained sections were viewed with DIC technique, the images illustrated an irregular tissue surface possibly caused by cellular edema in cortical as well as subcortical areas (Fig. 1c,d). Phase contrast images also disclosed a white rim around dysmorphic neurons possibly corresponding to the astrocyte end-feet swelling (Fig. 1e).

**Figure 2:**
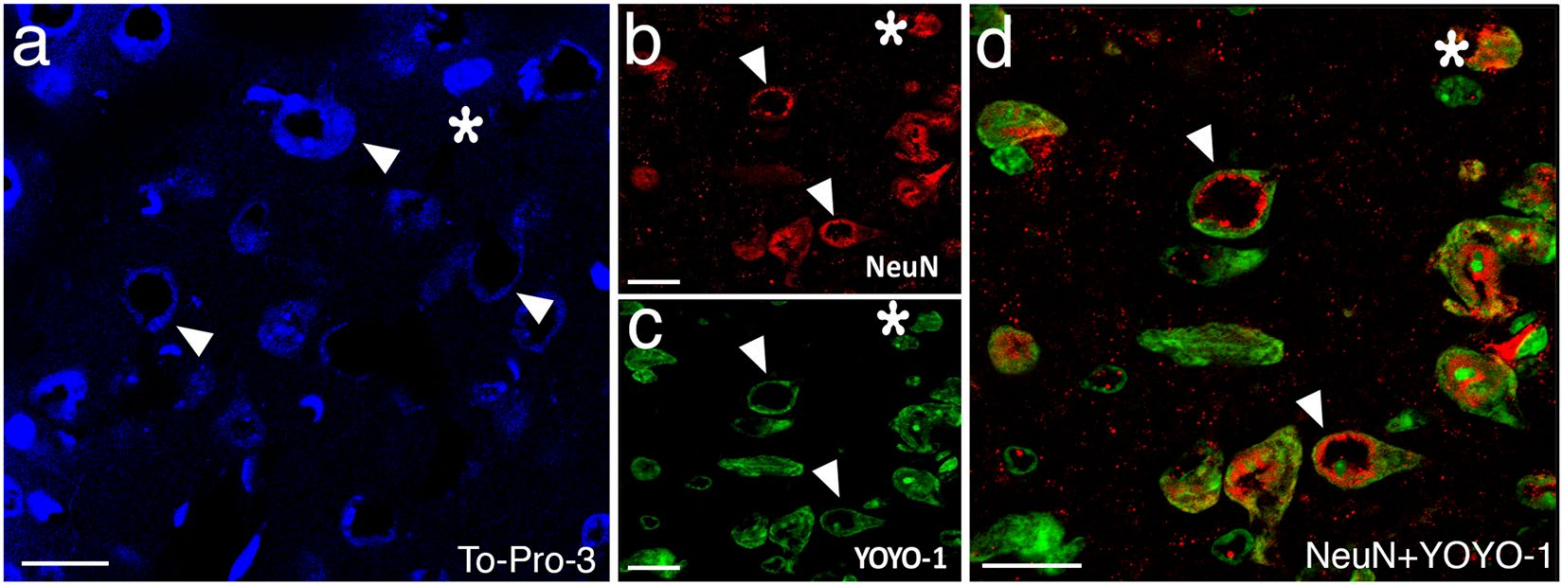
Donut-like staining of neurons in poorly perfused sections was also observed with other nuclear markers. Frozen sections exhibited abnormal (donut-like) staining pattern with nuclear stains TO-PRO-3 (triangles in **a**) similar to those seen with NeuN (triangles in **b**); and YOYO-1 (triangles in **c**). Merged image (**d**) illustrates the colocalization of abnormal nuclear staining with YOYO-1 and NeuN (triangles in **d**). Intact cells are shown by asterisks. Scale bars: 20 µm.

**Figure 3:**
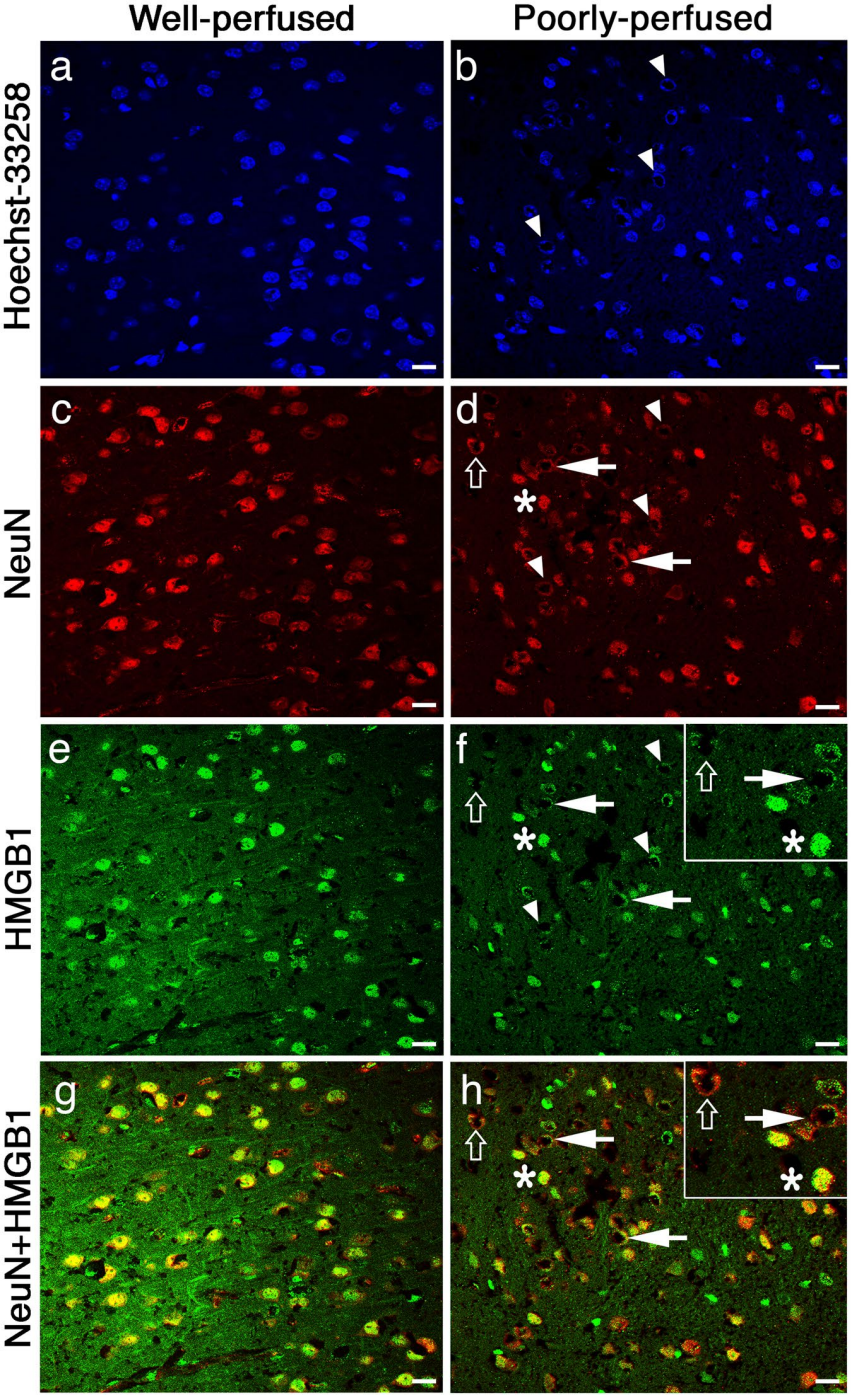
Dysmorphic neurons were stressed and released HMGB1 during poor perfusion. Unlike well-perfused brain sections (**a**,**c**,**e**,**g**), where HMGB1 immunolabeling was confined to dense intact nuclei (**e**,**g**), HMGB1 translocated to the cytoplasm or completely released to extracellular space from swollen nuclei in sections from poorly-perfused brains (**f**,**h**). These nuclei displayed donut-like staining pattern with Hoechst or NeuN (triangles in **b**,**d**). The “non-stressed” cells (asterisk) exhibiting normal nuclear HMGB1 immunolabeling (**f**,**h**) also showed normal nuclear staining pattern with Hoechst and NeuN (**b**,**d**). The merged images (**g**,**h**) illustrate intact neuronal nuclei (NeuN +, red signal) with nuclear HMGB1 staining (green signal, asterisk in **h**) and neuronal nuclei exhibiting donut-like staining with HMGB1 as well as NeuN (arrows in **h**) and donut-like neuronal nuclei that released most of its HMGB1 (open arrows in **h**). Inset in **h** illustrates neurons with nuclear HMGB1 (asterisk) and peripheralized nuclear HMGB1 staining (arrow) as well as weak, residual HMGB1 immunolabeling (open arrow) at higher magnification. Inset in **f** illustrates weak HMGB1 immunolabeling in dysmorphic neurons unlike strong nuclear staining in neighboring non-stressed neurons. Non-neuronal (NeuN−) cells exhibit strong nuclear HMGB1 staining (green nuclei in **h**). Images were taken from parietal cortex. Scale bars: 20 µm.

In unambiguously verified poorly perfused brain sections, we found out a previously unknown, instant morphological hallmark of neuronal injury; nuclear enlargement with peripheralization of chromatin that created a characteristic nuclear staining pattern. The center of the nuclei was not appreciably stained with any of the nuclear stains, YOYO-1, TO-PRO-3, Hoechst-33258 or the neuronal nuclear marker NeuN; creating a donut-like appearance in frozen sections prepared from poorly-perfused brains (Figs 1f, 2 and 3a–d). Swelling of the nucleus may have caused this staining pattern because the mean nuclear radius in layer 3 of parietal cortex was increased to 14 ± 0 µm (N = 240) as detected by Hoechst-33258 staining in poorly-perfused brains unlike 8 ± 1 µm (N = 240) normal nuclear size in well-perfused brains (p < 0.01). The donut-like cells were all NeuN-positive, indicating that they were neurons. In addition to conventional nuclear markers, we also evaluated these sections for HMGB1 immunoreactivity (Fig. 3e–h) because translocation of this nuclear protein to cytoplasm or total release from the cell is an indicator of cellular stress. Indeed, 50 ± 3% (N = 168) of the donut-like stained neurons showed loss of HMGB1 immunostaining possibly due to its massive release out of cell (extracellular translocation), whereas 7 ± 3% displayed HMGB1 translocation from nucleus to cytoplasm (cytoplasmic immunostaining), confirming that these cells had been under metabolic stress before being fixed. Interestingly, residual nuclear HMGB1 staining also exhibited a donut-like nuclear staining pattern (Fig. 3f), suggesting that nuclear chromatin was displaced toward the periphery of the nucleus (see EM findings below) as also implied by Hoechst, YOYO-1, TO-PRO-3 and NeuN staining, all of which label proteins associated with chromatin^14–17^.

**Figure 4:**
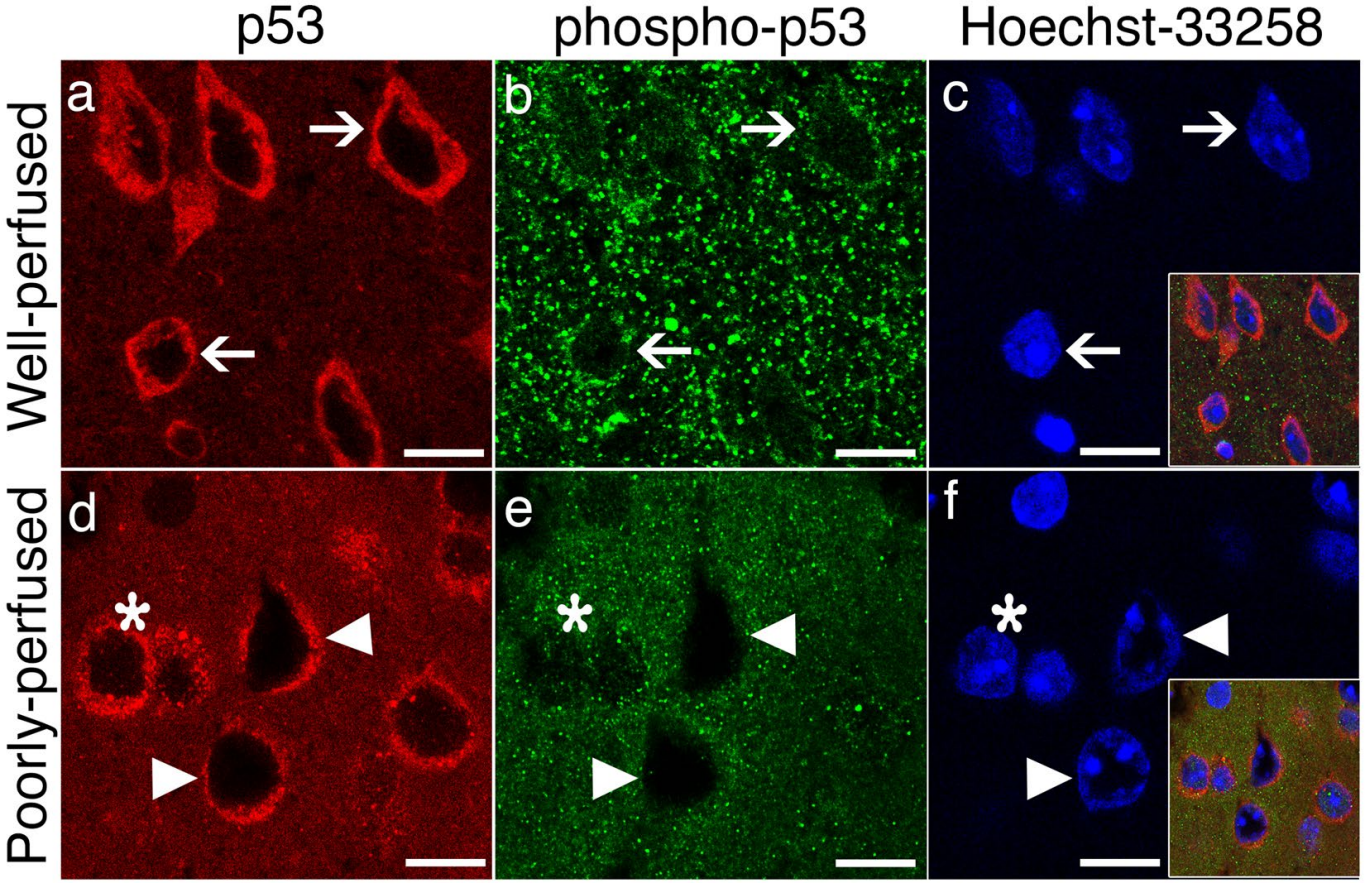
Poor perfusion instantly activated p53-mediated death signaling. In well-perfused brains (upper row), p53 immunolabeling (red) exhibits a predominantly cytoplasmic and diffuse staining (arrows, **a**), whereas anti-serine15-phospho-p53 antibodies weakly immunostained the nuclei (arrows, green, **b**). (**c**) Illustrates the intact nuclei identified by Hoechst staining (arrows, blue). The inset is the merged image of (**a**,**b**,**c**). In poorly perfused brain sections (lower row), p53 is still predominantly expressed in the cytoplasm but assumed a granular pattern (red, **d**). Triangles point at the swollen donut-like stained nuclei (**d–f**). These nuclei were not immunolabeled with phospho-p53 antibodies (green, **e**), whereas other intact nuclei preserved phospho-p53 immunopositivity (*). Coarse granular cytoplasmic phospho-p53 immunostaining (**b**) was replaced by a fine granular pattern in poorly perfused brain sections (**e**,**f**) illustrates the nuclei identified by Hoechst staining (blue). The inset is the merged image of (**d**,**e** and **f**). Scale bars: 10 µm.

**Figure 5:**
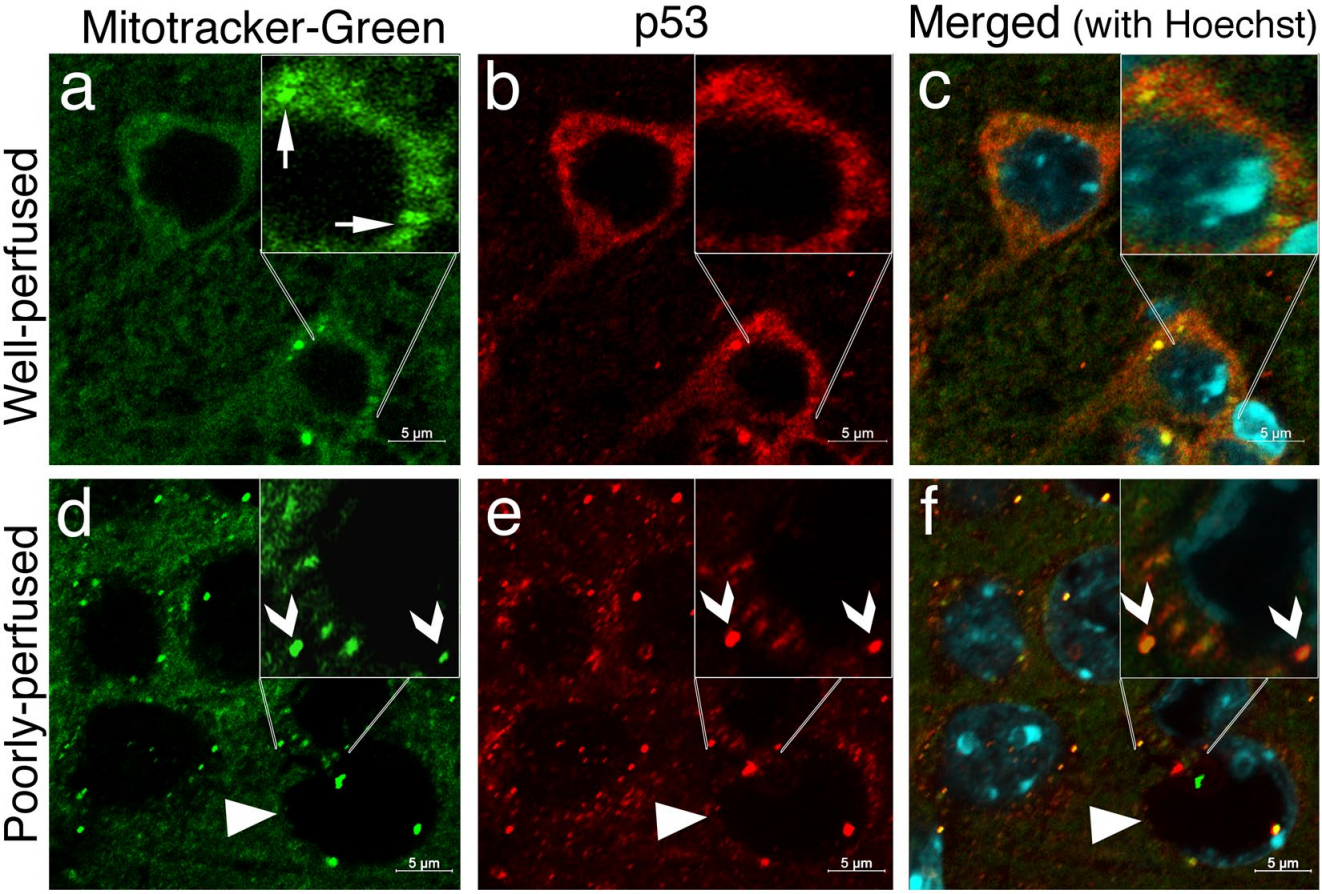
p53 translocates to mitochondria in poorly perfused brain sections. In well-perfused brains (upper row), p53 immunolabeling (red) exhibits a predominantly diffuse cytoplasmic staining (**b**) and occasional colocalization with mitochondria visualized with mitotracker-green (arrows, green, **a)**. (**c**) illustrates the nuclei identified by Hoechst staining (blue) on a merged image of (**a** and **b**). In poorly perfused brain sections (lower row), p53 immunopositive granules (arrowheads, red, **e**) sharply colocalize with mitochondria (arrowheads, green, **d**). (**f**) Illustrates the nuclei identified by Hoechst staining (blue) on the merged image of (**d** and **e**). Triangles point at a swollen donut-like stained nucleus. Insets show magnified images. Images were taken at 0.2 µm thickness by laser scanning confocal microscope. Scale bars: 5 µm.

We also assessed whether these injured-neurons had already activated cell death pathways within the few minutes before fixation. For this, we used p53 immunochemistry because, when neurons are severely stressed, cytosolic p53 translocates to mitochondria to initiate the intrinsic cell death pathway^18^ although this phenomenon has never been examined so early as in our study (Fig. 4). In well-perfused brains, p53 immunolabeling exhibited a predominantly cytoplasmic and diffuse staining (Fig. 4a). Nuclear staining was very weak. In poorly perfused brain sections, p53 was still predominantly expressed in the cytoplasm but assumed a granular pattern in addition to diffuse staining (Fig. 4d). These p53-immunopositive granules were colocalized with mitochondria visualized by mitotracker-green (Fig. 5). The weak nuclear staining persisted except in donut-like stained nuclei. Similarly, these nuclei were not immunolabeled with anti-serine15-phospho-p53 antibodies, which weakly immunostained the nucleus in well-perfused sections (Fig. 4b). Coarse granular cytoplasmic phospho-p53 immunostaining changed to a fine granular pattern in poorly perfused brain sections Fig. 4b,e).

To assess which of the nuclear markers could predict the quality of the perfusion best, we quantified the number of abnormally-stained neurons in two cortical and one subcortical areas and compared the number of donut-like stained cells. The numbers detected in poorly-perfused brains were significantly higher than were they in well-perfused brains (p < 0.05, Table 1). NeuN labeling seemingly gave a higher ratio than other stains because it shows the proportion of neurons with donut-like stained nuclei to total number of neurons unlike other stains, which gives the ratio of donuts to the total nuclei including glial cells. Based on these data, we suggest a donut-like nuclear staining threshold of 2% for Hoechst and of 6% for NeuN labeling to differentiate between poorly and well-perfused brain sections.

**Table 1:** Quantification of donut-like stained nuclei in the brain.

	Brain areas	NeuN* (%)	Hoechst* (%)	HMGB1* (%)	Nissl* (%)
**Poorly-perfused brain**	parietal cortex	46 ± 2	31 ± 3	41 ± 5	36 ± 5
	hind- and forelimb areas of cortex	43 ± 5	34 ± 4	39 ± 6	32 ± 4
	preoptic area (medial and lateral)	55 ± 4	34 ± 4	32 ± 5	36 ± 2
**Well-perfused brain**	parietal cortex	5 ± 1	1 ± 0	2 ± 1	5 ± 0
	hind- and forelimb areas of cortex	6 ± 1	2 ± 1	3 ± 1	6 ± 1
	preoptic area (medial and lateral)	3 ± 1	2 ± 0	3 ± 1	5 ± 1

In one subcortical and two cortical areas the average percentage of abnormally stained donut-like nuclei are given for poorly-perfused (6 mice) and well-perfused (4 mice) brains with different markers. For Nissl staining, the numbers refer to the ratio of dysmorphic neurons surrounded by swollen astrocyte end-feet to total cell nuclei in the contralateral hemisphere embedded in paraffin. NeuN staining gives a higher ratio because it shows the proportion of donut-stained neurons to total number of neurons unlike other markers, which give the ratio of donuts to the total nuclei including glial cells. Data are expressed as mean (percentage) ± standard error of mean (SEM). *p < 0.05 when poorly- and well-perfused groups compared. Brain anatomical areas are defined at the level of the anterior commissure according to Garcia *et al*.^2^.

### Transmission and Scanning Electron Microscopy

**Figure 6:**
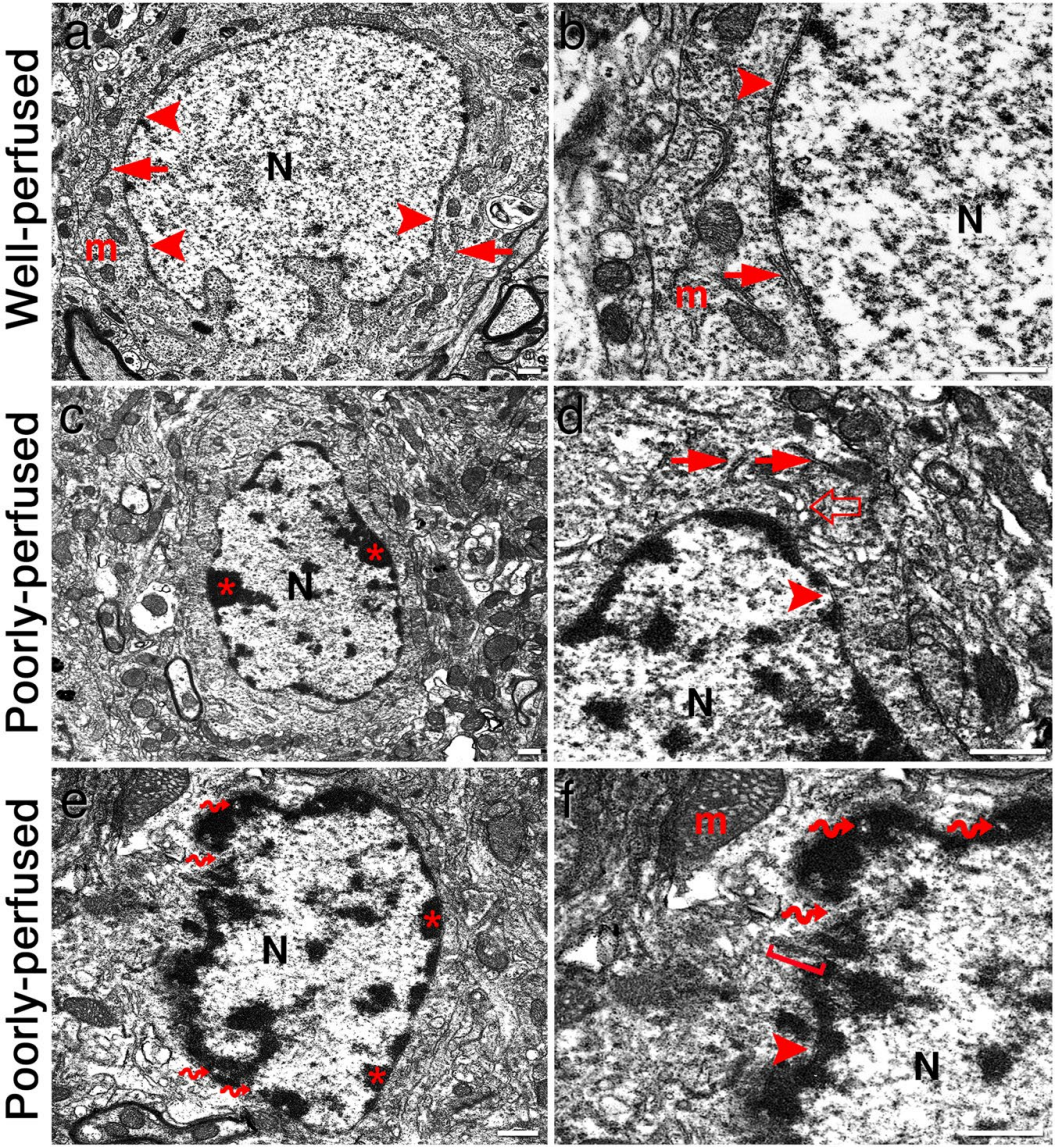
Representative TEM images taken from parietal cortex. An intact neuron in parietal cortex of a well-perfused brain with large, euchromatic nucleus (**N**) (**a**). Nuclear double membrane (arrowheads in **a**,**b**,**d**,**f**) is intact and regular. Chromatin material spreads homogenously to all parts of the nucleus. Cytoplasm has abundant free ribosomes and polyribosomes, and several well-preserved mitochondria (**m**) and sections of rough endoplasmic reticulum (RER) (arrows in **a**,**b**,**d**). Cytoskeletal filaments are also well preserved. In higher magnification of the same cell (**b**), we noted that double nuclear membrane is continuous and chromatin spreads homogenously. In the cytoplasm, mitochondria, RER and rosette-like polyribosomes are well preserved. In the same area of the cortex from a poorly-perfused mouse brain (**c**), the neuronal cytoplasm is moderately shrunken and condensed compared to the well-perfused brains. Clarity of the mitochondria, polyribosomes and RER was not perfect unlike in well-preserved neurons. Chromatin material (*) is peripheralized and accumulate under the nuclear membrane. At higher magnification of the same cell (**d**), the nuclear double membrane is intact and RER sacs are found enlarged (open arrow) compared to the RER sacs at the periphery of the cell (arrows). Chromatin clumping and margination under the nuclear membrane are evident. In a neuron from a poorly-perfused cortex (**e**), several nuclear pores (curved arrows) as well as chromatin clumping (*) and margination under the nuclear envelope are clearly visible. The nuclear envelope is irregularly folded. In higher magnification of the same cell (**f**), a RER sac (bracket) is found inserted through the enlarged nuclear pore, possibly compensating the expansion of the nuclear membrane due to swelling. Scale bars: 500 µm.

Examination of poorly perfused brains with TEM (n = 3) showed chromatin clumping and margination under the nuclear envelope in neurons unlike well-fixed ones (Fig. 6a–d). The nuclear double membrane was intact but irregularly folded (Fig. 6d). Some of the nuclear pores were noticeably enlarged and a rough endoplasmic reticulum (RER) sac was inserted through the enlarged nuclear pore, possibly compensating the expansion of the nuclear membrane due to swelling (Fig. 6e,f). In addition to these striking nuclear changes, some RER sacs around the nucleus were enlarged (Fig. 6d). Clarity of mitochondria, polyribosomes and RER were not fine as in well-perfused brain. In some neurons, the cytoplasm was moderately shrunken and condensed.

**Figure 7:**
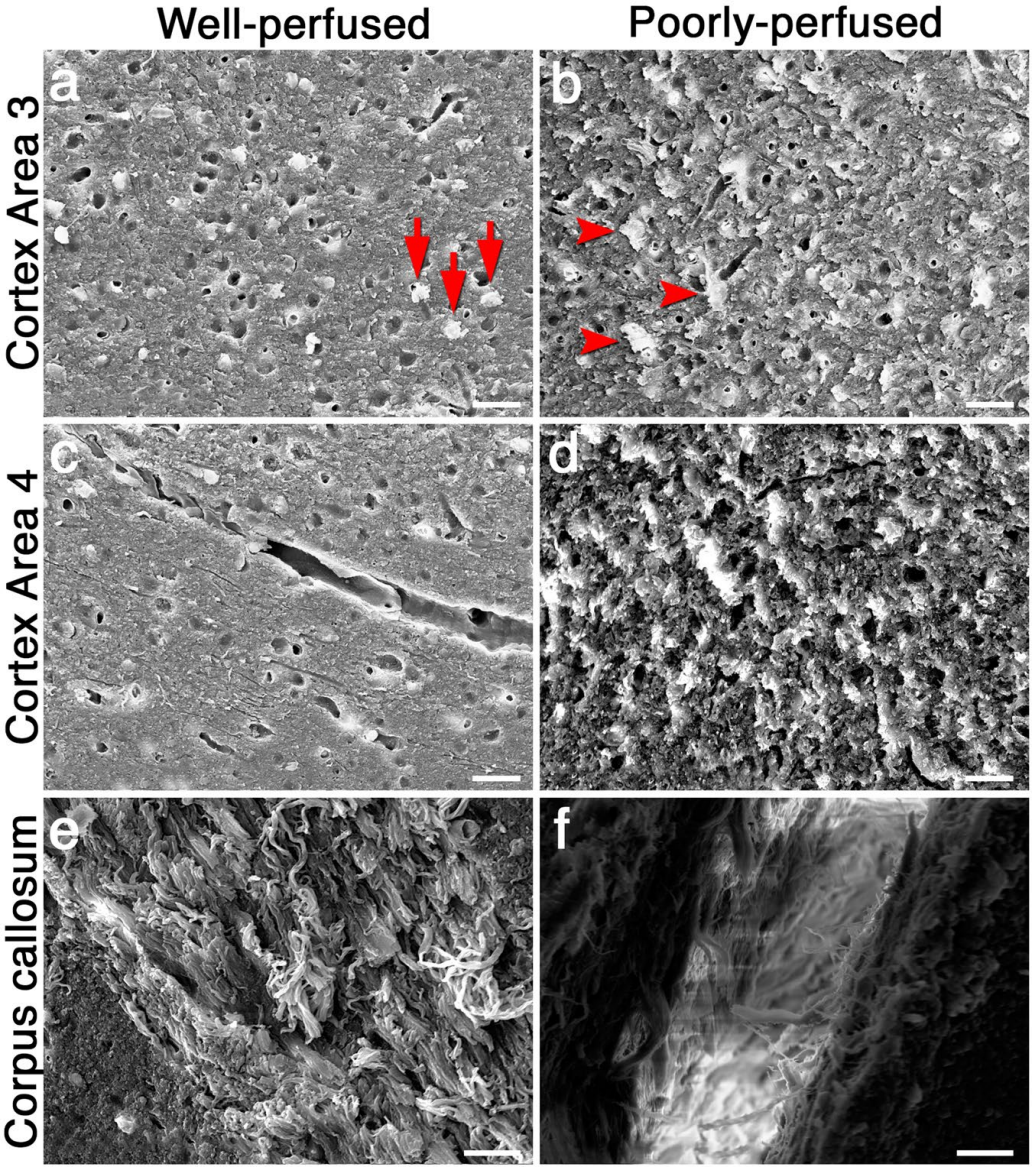
Representative SEM images taken from cortex 3, 4 and corpus callosum. A well-perfused section from cortex 3 displays relatively round neuron somas (arrows in **a**), whereas dysmorphic and swollen neural somas are seen in poorly-perfused samples (arrowheads in **b**). Integrity of the intercellular tissue is also disrupted in poorly-perfused sections (**b**). Fine structure of blood vessels in cortex 4 is clearly discernable in well-perfused samples (**c**) unlike poorly-perfused samples (**d**). Neuronal fibers are well-preserved in well-perfused sections taken from corpus callosum (**e**), whereas intense electron discharges emerge as a result of poor perfusion (**f**). Scale bars: 20 µm.

Scanning electron microscopic (SEM) thin sections are known to be highly sensitive to inadequate perfusion. The presence of donuts in fluorescent labeled sections was associated with an irregular tissue surface in SEM sections, suggesting that cellular swelling was not limited to neuronal nuclei (Fig. 7). SEM analysis of poorly-perfused brain sections also demonstrated a series of atypical structural alterations encompassing the entire brain tissue. Figure 7 shows representative images from three different anatomic regions in well-perfused and poorly perfused brain sections. Dysmorphic and swollen neuron somas were observed in poorly perfused samples embedded in a disintegrated intercellular matrix (Fig. 7b). Fine structure and continuity of blood vessels were lost in poorly perfused samples (Fig. 7d). Neurites, noticed as fine fibers, could not be visualized because of intense electron discharges as a result of poor perfusion-fixation (Fig. 7f).

### Leptomycin B and metformin inhibit nuclear swelling in poorly-perfused brains

**Figure 8:**
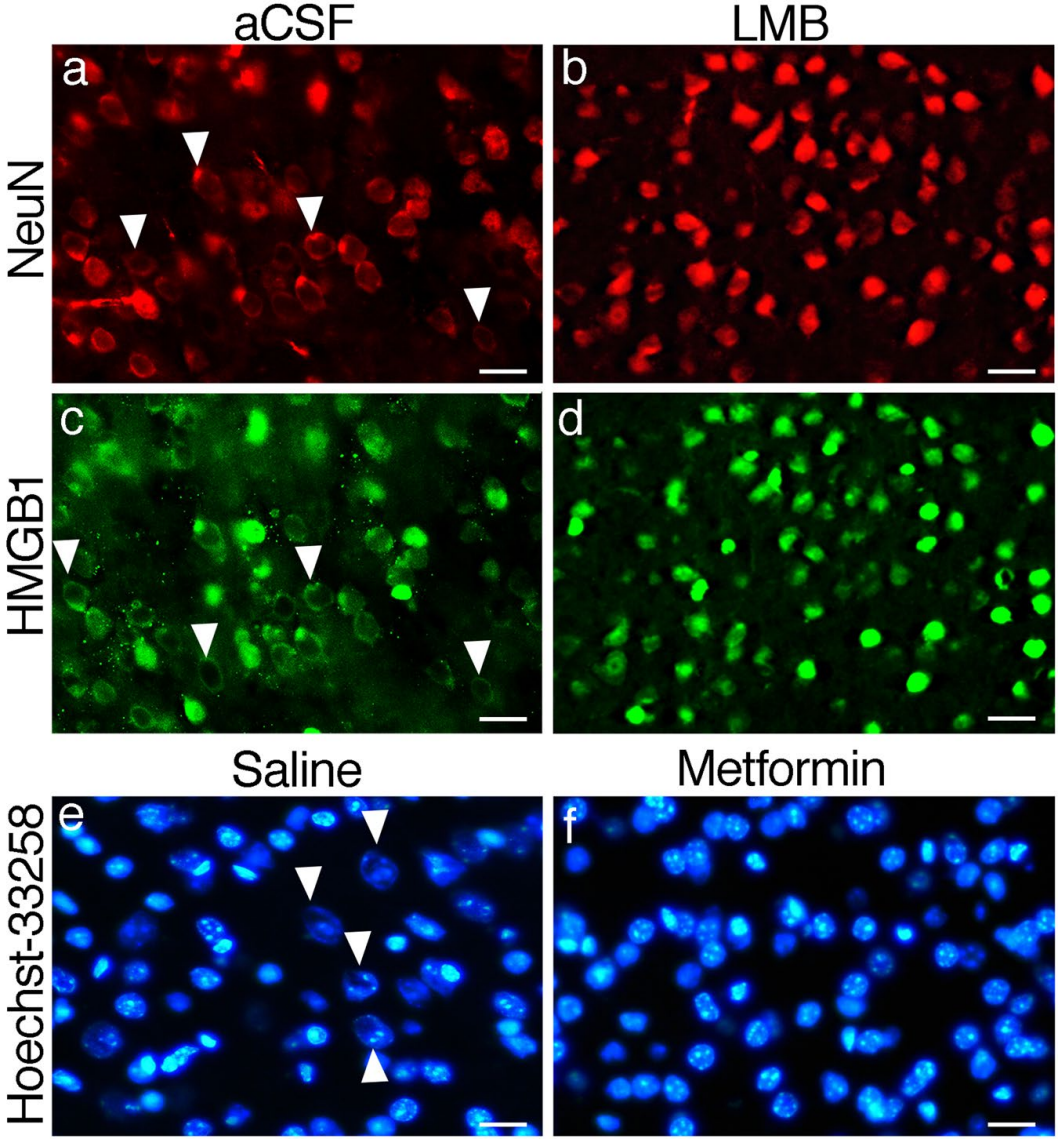
Donut-like nuclear staining and nuclear swelling were prevented by nuclear pore complex inhibitors. Nuclear transporter exportin-1 inhibitor leptomycin B prevented donut-like nuclear staining when injected intracortically (LMB, 3 ng/µL) to the right hemisphere (**b**,**d**) in a poorly-perfused mouse. The contralateral homolog cortex of the same animal was injected with the vehicle (aCSF) and exhibits several donut-like NeuN-positive nuclei (triangles) also stains with a donut-like pattern by HMGB1 (**a**,**c**). These abnormal patterns are absent in the LMB injected hemisphere (**b**,**d**). NeuN and HMGB1 were labeled on the same brain section. Nuclear pore complex inhibitor metformin (intracortical, 3 µg/µL) also prevents donut-like nuclear staining. Contralateral cortex injected with vehicle (saline) exhibits donut-like nuclear staining with Hoechst (triangles**, e**) whereas this pattern is totally prevented in the metformin-injected cortex of the poorly-perfused brains (**f**). Epifluorescent images with scale bars: 20 µm.

Both leptomycin B (LMB) and metformin inhibited nuclear swelling in poorly-perfused brains when administered 30 min before perfusing the animal. We adjusted the dose of each agent such that the protective effect of the drug was observed only at the injection site to be able to compare this area with the rest of the brain that exhibits the signs of poor perfusion. Since higher doses reached effective concentrations across both hemispheres, they protected the whole brain; hence, it was hard to make sure whether the brain was well perfused or protected by the inhibitors of the nuclear pore complex. For this, we injected 3 ng/µl of LMB or 3 µg/µl of metformin intracortically to the area 4 of the cortex. No donut-like nuclear staining pattern was observed with Hoechst-33258, NeuN or HMGB1 at the injection site of both inhibitors (Fig. 8b,d,f). In the contralateral hemisphere where only vehicle was injected, we found that 16 ± 0% of the nuclei (n = 257) in LMB group and 16 ± 2% of the nuclei (n = 380) in metformin group, exhibited donut-like staining with Hoechst, being above the threshold detected in well perfused brains (Fig. 8e). Donut-like staining was also observed with NeuN and HMGB1 in vehicle-injected hemisphere (Fig. 8a,c). The nuclear radius, measured at the parietal cortex layer 3 with Hoechst stain, dropped to 7 ± 1 µm (n = 240) and 9 ± 2 µm (n = 240) (for LMB and metformin, respectively) at the injection site when compared to vehicle injected contralateral homolog area (13 ± 1 µm (n = 240) and 12 ± 1 µm (n = 240) for LMB and metformin, respectively; p < 0.05) (Fig. 8e,f).

## Discussion

We have found that neuronal injury caused by poor perfusion can easily be identified with several fluorescent markers, including commonly used nuclear labels Hoechst, YOYO-1 and TO-PRO-3 as well as the neuronal marker NeuN. Hoechst could be the most practical label to check the quality of perfusion-fixation because it can be readily applied as a mounting solution and does not interfere with green and red fluorescent labels, allowing further examination of the preparation if it is well perfused. Such a quality check before in depth examination of a microscopic slide is critical to avoid confounding effects of hypoperfusion/hypoxia on the biological phenomena of interest. By combining several microscopic techniques, we suggest that the donut-like stained nuclei correspond to swollen neuronal nuclei showing chromatin margination and are a clear indication that the brain suffered from hypoperfusion/hypoxia before completely being fixed. We propose a threshold of 6% for NeuN and 2% for Hoechst staining in the parietal cortex as the acceptable limit for optimal perfusion (Table 1).

Our findings also provide insight to some of the first steps of the complex death mechanisms in neurons and identify nuclear swelling with chromatin margination as an immediate reaction. The peripheralization of chromatin suggested by donut-like staining with nuclear markers or immunohistochemistry, was also directly confirmed with TEM. Considering that these changes have rapidly taken place within a few minutes before tissue fixation, it is likely that they might have been caused by the structural changes in connections of the nuclear envelope with cytoskeletal fibers and chromatin during nuclear swelling^19,20^. Donut-like appearance may have not been caused by slow and non-uniform fixation of nuclear proteins because several simultaneously fixed neighboring neuronal nuclei exhibited a normal chromatin distribution and, the nuclear pore inhibitors were able to prevent chromatin margination in the poorly fixed brains.

Intriguingly, although the cellular/nuclear swelling is known to be notoriously resistant to treatment, here, we found that inhibitors that act through the nuclear pore complex could prevent the early nuclear swelling. This suggests that the increase in nuclear radius, an early reaction to energy failure, could allow the expansion of the nuclear envelope without rupture (Fig. 6d) by recruiting membrane from the endoplasmic reticulum that is in continuum with the nuclear membrane as well as by expansion of the nuclear pores^13^ (Fig. 6e,f). Prevention of the nuclear swelling with metformin also suggests that the flux of solutes and water from cytoplasm to nucleus is mediated through the expanded nuclear pores^13^. This finding excludes the possibility that the observed nuclear changes could be secondary to an unidentified neurotrophic pathogen as also suggested by lack of similar changes in well-perfused brains and of any pathological changes that might suggest encephalitis. The agents we used act on different sites involved in import/export mechanisms; metformin acts through nuclear pore complex^21^, whereas LMB blocks exportin-1^22^. The findings with LMB suggest that HMGB1 and NeuN are released into cytoplasm by way of exportin-1 as reported for release of these proteins from nucleus to cytoplasm in an animal model of status epilepticus^22^. In addition to the role of nuclear pore complex in caspase-mediated apoptotic nuclear dismantling, it was suggested that their increased permeability might also facilitate the exchange between the nucleus and cytoplasm early during HeLa cell apoptosis^23^. Our observations point to the role of nuclear pores in neuronal death as well, and warrant further research to understand the function of the nuclear pore complex and transporters in neuronal death mechanisms as well as the possible neuroprotection that could be obtained with their inhibitors. Indeed, a recent study reported that calpain-dependent degradation of nucleoporins contributed to motor neuron death in a mouse model of chronic excitotoxicity^24^. Several conditions that can potentially cause acute neuronal injury such as brain surgery, cerebrovascular interventions can effectively be prevented by targeting these immediate nuclear changes before activation of the intrinsic death pathway or HMGB1 release from nucleus. Indeed, a recent study has reported prevention of cell death after pilocarpin-induced status epilepticus with LMB pre-treatment in rats^22^. Furthermore, in addition to acute injury, mechanisms involving the nuclear pore complex may play critical roles in neurodegenerative diseases too. Metformin, a widely used anti-diabetic drug, has recently been reported to reduce cancer risk possibly by inhibiting nuclear pore complex^21^. Metformin use in diabetes has also been suggested to be neuroprotective against Alzheimer and Parkinson diseases, which might involve a similar mechanism, opening new and exciting opportunities for neuroprotective drug repurposing and development^25^. Of note, the effect of metformin cannot solely be secondary to its anti-diabetic action, reducing vascular and metabolic complications of diabetes because it stands out among other anti-diabetic drugs with regard to neuroprotection although the clinical data are yet not so compelling.

In addition to structural changes, it was also possible to identify the immediate molecular responses to neuronal injury in brain sections subjected to brief but severe hypoperfusion/hypoxia before complete fixation. Similar to rapid translocation of HMGB1 from nucleus, we found that p53, a nuclear transcription factor that it is also highly expressed in the cytoplasm and translocates to the outer mitochondrial membrane in response to stress, instantly translocated to mitochondria during hypoperfusion/hypoxia before fixation. Phospho-p53 immunostaining also acquired a fine granular pattern in the cytoplasm, consistent with mitochondrial translocation. Targeting p53 to the mitochondrial outer membrane has been shown to be sufficient to promote apoptosis in neurons several hours after global ischemia, neonatal hypoxia-ischemia, proteasome inhibition and DNA damage^26–29^ notwithstanding some reservations about its significance in neuronal death^18^. Our findings corroborate these positive reports and additionally suggest that mitochondrial p53 translocation can be an *immediate* response to neuronal injury *in vivo* as demonstrated here for the first time. The neuronal stress during perfusion-fixation was too short to induce transcriptional p53 activity; instead, phospho-p53 and p53, both of which are weakly expressed in normal nuclei, were lost from donut-like nuclei, suggesting their translocation to cytoplasm.

The literature investigating the effect of poor perfusion on brain tissue is scarce and this important subject seems have not been studied thoroughly. The reported electron microscopic signs of poor perfusion-fixation include extracellular space enlargement, mitochondrial swelling and dendritic and astrocytic lamellar swelling^30,31^. It has been shown that extension of the perfusion-fixation time imitates ischemic stress and causes increases in the thickness of post synaptic densities in the CA1 region of hippocampus, layer III of cerebral cortex and Purkinje spines of cerebellar cortex due to NMDA receptor overactivation^32,33^. Moreover, staining with buffered osmium for electron microscopy after poor cardiac perfusion with aldehydes has been shown to be complicated by tissue shrinkage^30^. In an MRI study, fixed mouse brain magnetic resonance images showed gray–white matter contrast inversion when the fixation time was too short or fixative concentration was too low^34^.

We should note that long perfusion time exceeding one minute with heparinized saline preceding paraformaldehyde (PFA) perfusion-fixation, can also lead to hypoxic/ischemic changes because the tissue is still viable and not fixed as the fixative solution has yet not been introduced. Although some authors have suggested to skip the flushing out of the blood because of this drawback^35^, this is usually not preferable because blood clots and IgG remaining in the tissue may interfere with immunohistochemistry. Generally, 3–5 ml/min flow rates are suggested for perfusion of the mouse in brain studies^35^. In daily laboratory practice however, despite the fact that a correct perfusion flow rate is used, factors such as unsuitable localization of needle in the left ventricle, premature cessation of effective cardiac contractions and air bubbles introduced can still lead to inadequate perfusion. A rapid fixation is especially mandatory for the brain tissue as neurons are highly sensitive to hypoxia as well as hypoperfusion and, readily develop biochemical and morphological changes that can confound the targeted processes to be investigated.

In conclusion, donut-like staining of swollen neuronal nuclei can easily be detected with commonly used nuclear stains such as Hoechst and, be used as a biomarker of poor fixation on frozen brain sections. The morphological correlate of this staining pattern, neuronal nuclear swelling with chromatin margination appears to be a very early sign of metabolic compromise, which can be prevented by inhibitors of the nuclear pore complex. The latter observation may open new venues in cell death mechanisms and neuroprotection.

## Materials and Methods

### Animals

All animal experiments were performed in accordance with the institutional guidelines and regulations, and were approved by Hacettepe University Animal Experiments Ethics Committee (2017/07-3). A total number of 42 male and female Swiss albino mice (25 to 35 gr) were used; 9 in poorly-perfused, 7 in well-perfused groups for fluorescent labeling studies, others for leptomycin-B and metformin-treated groups and electron microscopy studies. Mice were obtained from the Animal Breeding and Housing Facility of Hacettepe University (certified by the Ministry of Food, Agriculture and Livestock). They were all healthy and free of infection. All mice were housed under a fixed 12-hour light/12-hour dark cycle with ad libitum access to food and water.

### Anesthesia and Perfusion

Naive mice were anesthetized with intraperitoneal injection of 1 mg/g chloral hydrate. After obtaining deep anesthesia, mice were positioned supine on the perfusion set-up. The ventral side of the body was disinfected with 70% ethanol. Sternum and the overlying skin and muscles were incised. Muscles were cut along the ribs, the diaphragm was separated from the chest wall, the rib cage was caudo-rostrally dissected and the sternum was lifted. The perfusion pump (MasterFlex console drive, Cole-Parmer Instrument Company, model 77800-60) was made ready by flushing its tubing with heparinized phosphate buffered saline (20 units/ml)^36^. A butterfly catheter with a 25 gauge perfusion needle was inserted into the left ventricle through the apex and the right atrial appendix was cut. After heparinized saline flushing, first, the liver and then the other organs turned pale. Subsequently, the heparinized saline perfusate was switched to 4% PFA without creating air bubbles and perfusion continued until the body became stiff. The mouse was decapitated with a large surgical pair of scissors and, after removing the skull; the brain was removed with a spatula inserted on the ventral side of the brain. The extracted brain was immersed into 4% PFA and kept at 4 °C. After approximately 24 hours of incubation in 4% PFA, the brains were cut sagittal into two halves; one half was kept in 30% sucrose solution for cryoprotection and fluorescence labeling, and the other half was prepared for Nissl and hematoxylin/eosin staining in order to be able to compare well-known hypoxic/hypoperfusion-induced changes in Nissl and hematoxylin/eosin with fluorescent staining of frozen sections. The procedures followed for scanning and transmission electron microscopy are given below in relevant subsections.

### Poor vs. Well Perfusion-fixation

Defining factors for well or poor perfusion are the total time for initial stage of the procedure including opening the chest and perfusion of heparinized saline and flow rate of the perfusates. In one group of mice (n = 7), the time measured from the first cut of the diaphragm until the end of heparinized saline perfusion was less than 2 minutes, of which 1 minute elapsed with heparinized saline infusion. At this point, perfusion was switched to 4% PFA for fixation. In this group, the flow rate was less than 3 mL/min in 3 mice and 3–4.5 mL/min in 4 mice. In another group (n = 9), the time between the first diaphragm cut and the start of 4% PFA infusion was extended to 4–8 minutes (2–3 minutes elapsed before starting heparinized saline and 2–5 minutes during heparinized saline perfusion). The flow rate also varied in this group as less than 3 mL/min in 5 mice and 3–4.5 mL/min in 4 mice.

### Fluorescent labeling of frozen sections

Twenty µm-thick coronal sections were cut on a freezing cryostat. Sections obtained were cryoprotected in 30% sucrose solution for two days. Sections were then immunolabeled with mouse monoclonal NeuN (1:200, Chemicon), rabbit polyclonal HMGB1 (1:200, Abcam), mouse monoclonal p53 (1:100, Santa Cruz) and rabbit polyclonal phospho-serine15-p53 antibody (1:100, Abcam), followed by secondary labeling with goat anti-mouse Cy3 (1:200, Jackson Immunoresearch) or goat anti-rabbit Cy2 antibody (1:200, Jackson Immunoresearch). In order to increase signal from poorly fixed sections, we used EDTA antigen retrieval (1 mM) at 95 °C for 10 minutes and added 0.3 M glycin to blocking solution to decrease non-specific staining. Sections were mounted with medium containing 1 µL/mL of Hoechst-33258 or YOYO-1 or TO-PRO-3 (Thermo Fisher Scientific). Sections were examined under a fluorescent microscope (Nikon E600) at different magnifications with appropriate filter settings. Representative sections were also imaged under laser scanning confocal microscope (Carl Zeiss LSM 510 and LSM 880 system equipped with fast airy scan detector, or Leica SP8) and with differential interference microscopy (DIC) (Carl Zeiss Axioimager M1).

### Histochemical Stainings

Paraffin blocks were deparaffinized in an incubator at 62 °C for one hour. They were immersed in xylene for 10 minutes and then rehydrated in 100%, 90%, and 70% ethanol solutions for three minutes in each. For Nissl, sections were washed with running distilled water and transferred into cresyl violet stain for 20 seconds. At this point, sections were checked under light microscope, if the color was strong enough to be visible under light microscopy, they were dried in oven for a few seconds and put in xylene and mounted with Entellan (Merck KGaA). For Hematoxylin and Eosin staining, the FD hematoxylin solution and FD eosin Y solution (FD Neurotechnologies; Catonsville, MD) were applied as described by the manufacturer.

### Scanning Electron Microscopy

From 3 poorly-perfused and 2 well-perfused mice, 2 to 3 mm-thick sagittal brain slices were cut manually (n = 6) to process for scanning electron microscopic (SEM) observations. Slices were initially washed with Sorenson phosphate buffer (0.1 M) for 15 minutes, fixed in 2.5% glutaraldehyde for 1 hour at room temperature, washed again and postfixed with osmium tetraoxide (1%) for 1 hour. Slides were then transferred to washing solution in Sorenson phosphate buffer and dehydrated in graded series of ethanol (15 minutes each). The slices were transferred to acetone and then dried in critical point dryer using acetone and CO_2_. Dried tissue slices were then placed on aluminum grids using carbon cement coated with gold-palladium (15 nm thickness) and viewed using Leo 438 VP SEM by secondary electron detector at 20 kEV.

### Transmission Electron Microscopy

After perfusion-fixation (3 poorly-perfused and 2 well-perfused mice) with the primary fixative, extracted brain tissues were excised to 1 mm thick slices and placed in 2.5% gluteraldehyde and 1% paraformaldehyde in 0.2 M phosphate buffer (pH 7.4) at room temperature for 2 hours. After two rinses in 0.1 M phosphate buffer, samples were placed in 1% osmium tetroxide in the same buffer for 1 hour at room temperature. Samples were subsequently rinsed two times in 0.1 M phosphate buffer and they were dehydrated by being passed through graded ethanol series and then embedded in Araldite CY-212. Semi-thin sections (0.5 µm) were cut with glass knives and stained with 1% toluidine blue O in 1% sodium borate. Ultrathin sections (90 nm) were cut with a diamond knife, stained with uranyl acetate and lead citrate and, examined using Leo 906 E (80 kV, Oberkohen-Germany) transmission electron microscope.

### Leptomycin B and Metformin injections

In order to investigate the role of nuclear pore complex in poor perfusion-induced nuclear swelling, different concentrations (3, 15 and 30 ng/µL in 1 µL of 10% methanol dissolved in artificial CSF) of leptomycin B (LMB; Sigma Aldrich L2913) was injected intra-cortically (i.c.) to the right parietal cortex of 3 mice to find out the optimal drug dosage. An equal volume of 10% methanol in artificial CSF (aCSF) was injected i.c. to the left homologous cortex of the same animal as control. The i.c. injections were performed at 1 mm ventral, 0.5 mm posterior and 2 mm lateral to bregma. The mouse was sacrificed by poor transcardial perfusion 30 minutes after injections. After finding the optimal concentration (3 ng/µL), its effect was tested on 4 poorly-perfused mice brains. We also used metformin hydrochloride (ab146725, Abcam), another inhibitor of the nuclear pore complex, first at 3 different concentrations (1.5, 3 and 7.5 µg/1 µL saline) in 3 mice with the same protocol as LMB injections except that saline, the vehicle of metformin was injected i.c. to the left homologous cortex of the same animal as control. All injections were done 30 minutes before sacrificing with poor transcardial perfusion. After finding the optimal concentration (3 µg/µL) metformin’s effect was tested on 4 poorly-perfused mice brains.

### Cell counting and Statistical Analysis

Hoechst, NeuN or HMGB1-labeled cells were counted at 200x magnification in two non-overlapping cortical areas and one subcortical area on 20 µm-thick coronal frozen brain sections. Nissl-stained cells were counted on the homolog areas of contralateral hemisphere on 5 µm-thick paraffin-embedded brain sections. Nuclear diameters were directly measured under fluorescent microscope on Hoechst-stained sections from 3 different regions of the parietal cortex layer 3 in each mouse with NIS-Elements (Nikon, Japan) tool at 400x magnification. Data were expressed as mean and the standard error of mean (mean ± sem) in the text. Kruskal-Wallis test was used for comparing groups. Statistically significant data was further analyzed with Mann-Whitney U test. A p value < 0.05 was regarded as statistically significant.
